# Cellular Toxicity and Immunological Effects of Carbon-based Nanomaterials

**DOI:** 10.1186/s12989-019-0299-z

**Published:** 2019-04-11

**Authors:** Xia Yuan, Xiangxian Zhang, Lu Sun, Yuquan Wei, Xiawei Wei

**Affiliations:** Laboratory of Aging Research and Cancer Drug Target, State Key Laboratory of Biotherapy and Cancer Center, National Clinical Research Center for Geriatrics, West China Hospital, Sichuan University, No. 17, Block 3, Southern Renmin Road, Chengdu, Sichuan 610041 People’s Republic of China

**Keywords:** Cytotoxicity, Carbon nanomaterial, Macrophage, Immunological effects

## Abstract

**Background:**

Carbon nanomaterials are a growing family of materials featuring unique physicochemical properties, and their widespread application is accompanied by increasing human exposure.

**Main body:**

Considerable efforts have been made to characterize the potential toxicity of carbon nanomaterials in vitro and in vivo. Many studies have reported various toxicology profiles of carbon nanomaterials. The different results of the cytotoxicity of the carbon-based materials might be related to the differences in the physicochemical properties or structures of carbon nanomaterials, types of target cells and methods of particle dispersion, *etc.* The reported cytotoxicity effects mainly included reactive oxygen species generation, DNA damage, lysosomal damage, mitochondrial dysfunction and eventual cell death via apoptosis or necrosis. Despite the cellular toxicity, the immunological effects of the carbon-based nanomaterials, such as the pulmonary macrophage activation and inflammation induced by carbon nanomaterials, have been thoroughly studied. The roles of carbon nanomaterials in activating different immune cells or inducing immunosuppression have also been addressed.

Conclusion: Here, we provide a review of the latest research findings on the toxicological profiles of carbon-based nanomaterials, highlighting both the cellular toxicities and immunological effects of carbon nanomaterials. This review provides information on the overall status, trends, and research needs for toxicological studies of carbon nanomaterials.

## Introduction

Nanotechnology has developed rapidly due to the expanding needs of many fields, including industry, agriculture, medicine and electronics [1]. Carbon-based nanomaterials represent the most promising products of nanotechnology, with fascinating properties that make them candidates for a variety of applications from drug delivery to electronics [2].

Carbon nanomaterials are a growing family of materials of different formations, such as black nanoparticles, fullerenes, carbon nanotubes (CNTs), fibres, and other related forms [3–8]. For instance, carbon black (CB) nanoparticles are traditional nanosized carbonaceous nanomaterials with a morphology consisting of grape-like aggregates of highly fused spherical particles, while carbon black is a quasi-graphitic form of nearly pure elemental carbon and presents as a major part of the ambient air pollution [9, 10]. C-fullerenes (C60) are characterized by symmetrical closed-cage structures that consist of 60 carbon atoms arranged in the shape of a soccer ball [10]. CNTs are fibrous materials with high aspect ratios and needle-like shapes, sharing physical similarities with asbestos fibres [11]. CNTs exist in two principle forms, namely, single-walled CNTs (SWCNTs) and multiwalled CNTs (MWCNTs), and can assume a wide variety of derived structures, such as horns, loops and peapods [12]. Single-walled carbon nanohorns (SWCNHs) are horn-shaped single-walled tubules with cone angles of approximately 20°. SWCNHs are synthesized by laser ablation and are essentially metal-free with high purity [13]. In addition, nanographite (NG), also called graphite nanoplatelets, is a one-atom-thick and two-dimensional sheet of sp2-bonded carbon atoms [14]. Each type of carbonaceous nanomaterial has a distinguishable shape.

In addition to manufactured nanoscale carbonaceous materials, naturally occurring carbonaceous particulates range from 1 to 100 nm in size exist. For instance, these materials exist in the emissions from certain combustion processes, such as the burning of methane and propane [12, 15]. Numerous studies have endeavoured to evaluate the health impact of occupational and environmental exposure to carbon nanomaterials [12, 16–19]. Studies on air pollution and mineral dust particles demonstrated that inhaled particles with a size < 100 nm caused lung injury through reactive oxygen species (ROS), cell damage and inflammation [20–23].

In vitro models have been developed for cytotoxicity studies to eliminate some variables in animal studies so that researchers could achieve better control over experimental conditions. A number of studies evaluated the harmful effects of carbon nanoparticles on various cell types, and as the first-line of defence against foreign particles, macrophages were mostly studied. Previous studies reported various toxic effects of carbon nanomaterials, including ROS generation, DNA damage, lysosomal damage, mitochondrial dysfunction and eventual cell death via apoptosis or necrosis [24–26]. The different results of the cytotoxicity of carbon-based materials might be related to many factors, such as the differences in the physicochemical properties or structures of carbon nanomaterials, types of target cells and methods of particle dispersion.

Despite the cellular toxicity, the immunological effects of carbon-based nanomaterials have been thoroughly studied. The immune system, as the first-line of defence in the human body, recognizes foreign agents and subsequently triggers immune responses [27]. The immune system is classified into innate and adaptive immune systems. The former functions as the first-line of defence, relying on the complement system and phagocytic cells at the forefront. Adaptive immunity proceeds through specific long-lasting mechanisms due to T and B lymphocytes [28, 29]. Regarding the immunological effects of carbon-based nanomaterials, the issues of pulmonary macrophage activation and inflammation induction are fully addressed. In addition, as nanoparticles (NPs) might enter the human body, including the lungs, the cardiovascular system, the liver and even the brain, through inhalation [20, 30], other adverse effects of carbon nanomaterials in vivo have also been characterized. The interactions of NPs with biological systems might generate toxicity related to their small size, large surface area and large surface reactivity [31, 32]. For example, relevant studies demonstrated that SWCNTs induced acute and chronic pulmonary pathologies [33], and systematic damage was detected in the blood chemistry [34], the liver [35] and the cardiovascular system [36]. Notably, ultrafine particles were capable of entering the bloodstream by crossing the alveolar-capillary barrier. After intravenous injection, SWCNTs can cross the blood-brain barrier and have been observed to enter the brain [37]. Moreover, special attention should be paid to the interaction of CNTs with components of the immune system. In the past decade, great effort has been made to shed light on the immunological properties of carbon-based nanomaterials [38, 39]; nevertheless, numerous questions remain because of the complexity of immune defense mechanisms.

## Main text

### Cellular Toxicity of Carbon-based Nanomaterials in Vitro

**Fig. 1:**
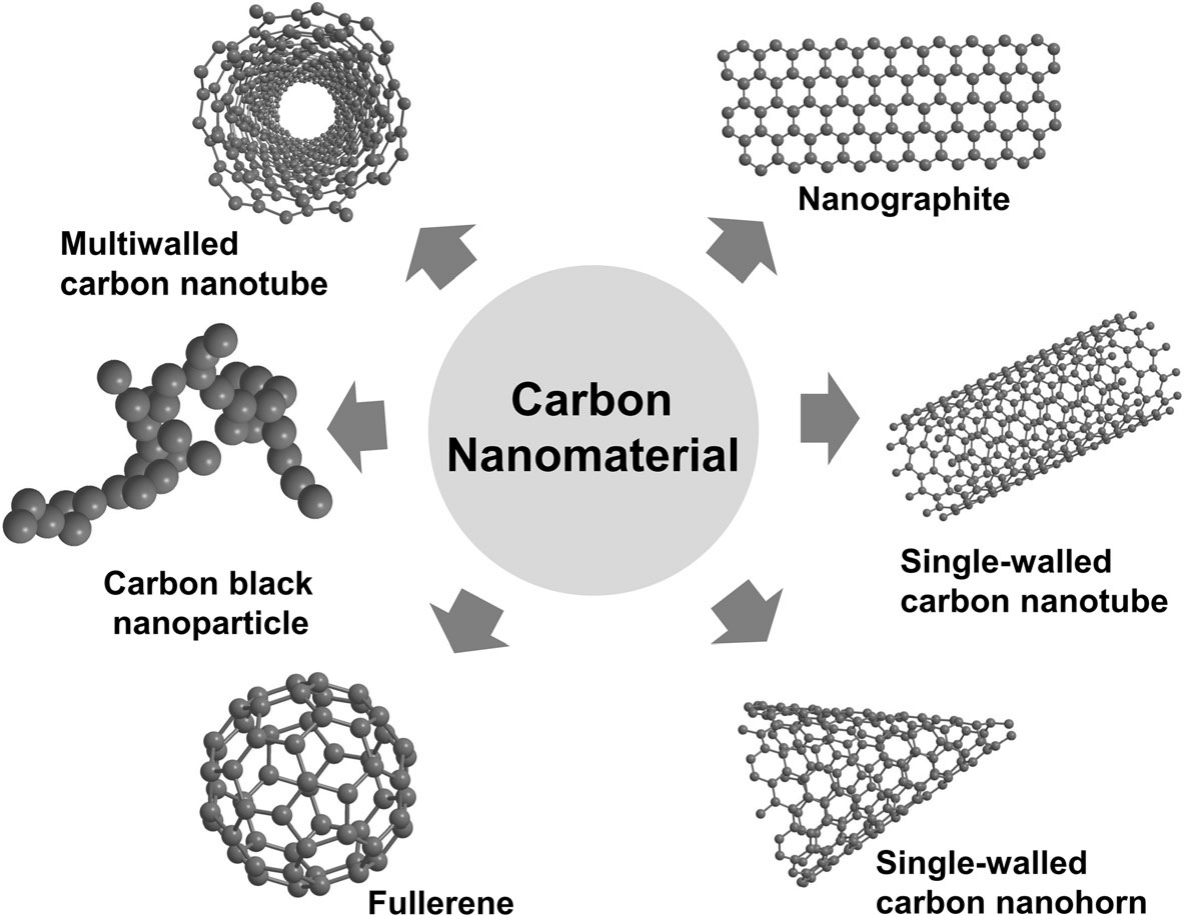
Various carbon-based nanomaterials were reported to induce cytotoxicity. Carbon nanotubes (CNTs) are thin carbon filaments with cylindrical structure that comprise single or multiple graphene sheets, termed as single-wall carbon nanotubes (SWCNTs) and multiwall carbon nanotubes (MWCNTs), respectively. C-fullerene is a carbon allotrope organized solely by 60 carbon atoms with a polygonal structure. Carbon black nanoparticle is a traditional nanosized carbon-based nanomaterial of diameter between 10 to 100 nm with three nanometric dimensions. Nanographite, also called graphite nanoplatelet, is a one-atom-thick and two-dimensional sheet of sp2-bonded carbon atoms. Single-walled carbon nanohorns (SWCNHs) are horn-shaped single-walled tubules with cone angles of approximately 20°

Here, we present a summary of current research efforts regarding the cytotoxicity of carbon nanomaterials in different cell types, including macrophages, epithelial cells and lymphocytes. The cytotoxicity of carbon nanomaterials will be discussed in terms of the following three aspects: different structures of carbon nanomaterials in cytotoxicity studies, the underlying mechanisms of cytotoxicity and some potential factors influencing detected cytotoxicity. Carbon nanomaterials with various structures in this review are presented in Fig. 1 and their cytotoxicity is summarized in Table 1.

**Table 1:** Examples of in vitro studies on cellular toxicity of various carbon-based nanomaterials

Types	Length	Diameter	Dose	Target cells	Cytotoxicity	Reference
Carbon nanotubes; Nanographite; Carbon black	1.5 μm; 4.5 μm; —	9.5 nm; 12 nm; —	15~120 μg/ml	RAW 264.7	LDH release, TNF-α production, ROS production	[9]
Pristine graphene	500-1000 nm	2~3 nm	5, 10, 20, 40, 80 and 100 μg/ ml	RAW 264.7	ROS increase, apoptosis by activation of the mitochondrial pathway, activation of the MAPKs (JNK, ERK and p38) and the TGF-beta-related signaling pathways	[14]
SWCNTs	—	—	0.78~200 μg/ml	HEK293 cell	Apoptosis and cell cycle arrest in G1.	[24]
Water-soluble fullerene	—	—	—	Human dermal broblasts, HepG2, neuronal human astrocytes	Lactate dehydrogenase release, cellular membrane disruption and lipid peroxidation	[25]
SWCNTs; MWCNTs; MWCNTs	—	1-2 nm; 10-20 nm; 30-50 nm	5~100 μg/ml	NR8383 cell	ROS generation and reduced cell viability.	[26]
Graphene oxides (GOs); Acid functionalized SWCNTs	—	500 nm; 355 nm	10~50 μg/ml	Peritoneal macrophages	LDH release, decreased autophagic degradation, lysosomal membrane destabilization	[40]
SWCNTs	150 nm	1~2 nm	0~50 μg/ml	Mouse peritoneal macrophages	Mitochondrial damage	[41]
MWCNT1; MWCNT2	13 μm; 5 μm	40~100 nm; 30 nm	0.625~10 μg/cm^2^	RAW 264.7	Mitochondrial activity reduction, LDH release	[42]
Aci- and tau-MWCNTs	5~10 μm	10~20 nm	0, 5, 20, 40, and 80 μg/ml	RAW 264.7	Apoptosis via mitochondrial pathway and scavenger receptor	[43]
SWCNT; MWCNT	1~5 μm; 1~2 μm	< 2 nm; 10~30 nm	30, 100 and 300 μg/ml	RAW 264.7	Cell death induced by SWCNT; no cell death induced by MWCNT	[44]
SWNTs; MWNT10; Fullerene	1 μm; 0.5~40 μm; —	1.4 nm; 10-20 nm; —	1.41~226.0 μg/cm^2^; 1.41~22.60 μg/cm^2^; 1.41~226.0 μg/cm^2^	Alveolar macrophage	Reduced cell viability	[45]
C-SWNTs; C60-fullerenes; Graphite particles	—	—	—	Human monocytes-derived macrophages	apoptosis/necrosis	[46]
Carbon black nanoparticles	175± 80 nm	20± 6 nm	30 μg/cm^2^	RAW264.7, human alveolar macrophages	Caspase 1 and IL-1β release, LDH release, plasma membrane disruption, pyroptosis	[47]
Fe@CNPs	—	—	50 and 400 μg/ ml	HEK293 and C33A cell	ROS generation and apoptosis	[48]
SWCNHs	400 nm	~100 nm	0.01~0.3 mg/ml	RAW 264.7	Apoptosis and necrosis associated with lysosomal membrane destabilization, ROS generation, inflammatory cytokines (TNF-α, IL-1β, and IL-6) release	[51]
MWCNTs	0.5-2 μm	< 8 nm; 20 30 nm; > 50 nm	100 μg/ml	3T3, RAW 264.7 and bronchiolar epithelial cells.	Cytotoxicity differing with particle sizes and cell types, reactive oxygen species generation, lysosomal membrane destabilization and mitochondrial permeability.	[52]
SWCNTs	—	0.8~2.0 nm	25 or 50 μg/cm^2^	Normal and malignant human mesothelial cells	ROS generation, increased cell death, enhanced DNA damage and H2AX phosphorylation, and activated PARP, AP-1, NF-κB, p38, and Akt	[55]
MWCNTs	< 1μm	9.5 nm	2.5~100 μg/ml	RAW264.7, A549	LDH release and oxidative stress	[57]
Carbon nanohorns	—	—	1~100 μg/ml	RAW 264.7	Reactive oxygen species generation and apoptosis lysosomal membrane permeabilization	[58]
Pristine-SWCNTs	—	—	1 μg/cm^2^	RAW264.7	Decreased cell viability and ATP production, increased ROS and NO production, activation of the MAP kinase pathway, increased levels of apoptosis- and autophagy-related proteins and ER stress-related proteins	[59]
Functionalized MWCNTs (tau-MWCNTs); Pristine MWCNTs (raw-MWCNTs);	300~600 nm; —	10~20 nm; —	0~ 80 μg/ml	RAW 264.7	Apoptosis related to mitochondrial injury, less toxicity induced by tau-MWCNTs	[60]
MWCNTs	—	—	20 μg/ml	Mature human monocyte-derived macrophage cells	Apoptosis and necrosis	[61]
Short MWCNTs; Long MWCNTs	0.6 μm; 20 μm	30.6 nm; 27.8 nm	10 μg/ml	Primary human alveolar macrophage	Reduced cell viability, ROS generation and inflammatory mediator release induced by long MWCNTs.	[64]
MWCNTs; Onion-like shell-shaped carbon nanoparticles;	~2 μm; —	10~15 nm; 50~100 nm	0~500 μg/ml	16HBE14o-	ROS generation, reduced cell viability	[70]
MWCNTs-COOH; MWCNTs-PEG	0.9 μm; 0.8 μm	24.6 nm; 27.3 nm	0~100 μg/ml	RAW 264.7 cells, primary rat peritoneal macrophages	Activation of oxidative stress-responsive pathways, such as p38 mitogen-activated protein kinases (MAPK) and nuclear factor (NF)-κB	[71]
Purified-MWCNT; COOH-MWCNT	1122 nm; 652 nm	—	1~50 μg/ml	Human alveolar macrophage	Reduced cell viability and increased inflammatory mediator (IL-1β and IL-8) release	[76]
Two types of functionalized carbon nanotubes (1,3-dipolar cycloaddition reaction and the oxidation- /amidation treatment)	—	—	1~10 μg/ml	Primary B lymphocytes, T lymphocytes, and peritoneal macrophages	Intake by B and T lymphocytes as well as macrophages in vitro without affecting cell viability.	[103]

#### Different structures of carbon nanomaterials used in cytotoxicity studies

##### Single-walled carbon nanotubes

It was reported that acid-functionalized SWCNTs (af-SWCNTs) displayed cytotoxicity in a concentration-dependent manner [40]. Another study showed that af-SWCNTs were engulfed by macrophages and then localized in lysosomes, leading to damaged mitochondrial function and inhibited phagocytic activity [41].

##### Multiwalled carbon nanotubes

Concerning MWCNT-induced cellular toxicity, available data are inconsistent. One study evaluated the toxic properties of two different MWCNT types, and neither of them induced apoptosis of RAW264.7 cells, as determined by caspase 3/7 activity [42]. In contrast, another study demonstrated that acid-treated MWCNTs (acid-MWCNTs) and taurine-functionalized MWCNTs (tau-MWCNTs) induced significant cell apoptosis and decreased cellular phagocytosis [43]. Different types of macrophages respond to MWCNTs distinctly. MWCNTs at any concentration from 3 to 30 μg/ml significantly induced cell death of murine bone marrow-derived dendritic cells (BMDCs), but for RAW264.7 cells, even 300 μg/ml MWCNTs presented no cytotoxicity [44].

##### Fullerene

It was reported that fullerene (C60) did not cause cytotoxicity in alveolar macrophages as observed through an MTT assay [45]. Similarly, C60 presented very low cytotoxicity against human macrophages and did not act as a biological inducer to elicit inflammatory reactions [46].

##### Carbon black nanoparticles

CB nanoparticles caused cell death in exposed human alveolar macrophages and RAW264.7 cells, characterized by a series of events including cell size enlargement, cell membrane rupture, caspase-1 activation, lactate dehydrogenase (LDH) leakage and IL-1β release [47]. Core/shell iron/carbon nanoparticles (Fe@CNPs) are applied in magnetic resonance imaging (MRI) and drug delivery. One study evaluated the cytotoxicity of variously functionalized Fe@CNPs (e.g., acrylic acid, pyrrolidone, primary amine and alkyl alcohol) in human embryonic kidney (HEK293) cells and human cervical carcinoma cells (C33A). Surface-modified Fe@CNPs triggered ROS generation to a similar degree, but only acrylic acid-functionalized Fe@CNPs induced cell death through the apoptotic pathway, suggesting that there is no association between ROS production and Fe@CNPs-induced cell apoptosis [48].

##### Nanographite

It was reported that pristine graphene induced typical cell death, including apoptosis and necrosis, in RAW 264.7 macrophages [14]. The toxicity of NG, CNTs and CB in RAW264.7 cells was compared through extracellular LDH release. Remarkable LDH release was observed only in the groups treated with CNT and NG at the highest dose, and NG produced stronger cellular toxicity than CNTs [9]. Due to the wide use of nanomaterials, many derivatives have been produced, and some of these derivatives have exhibited cellular toxicity [49, 50]. Wan et al. reported that graphene oxides (GO) exerted adverse effects on murine peritoneal macrophages [40].

##### Single-walled carbon nanohorns

A number of studies revealed the cytotoxicity of carbon nanomaterials under low-uptake conditions. Additionally, one study reported the response of RAW264.7 cells to high uptake of SWCNHs at 0.3 mg/ml; excess SWCNH uptake caused cell death, including both apoptotic and necrotic mechanisms [51].

#### The underlying mechanisms of cytotoxicity

##### ROS

MWCNTs induced cell apoptosis of 3T3 fibroblasts, bronchial epithelial cells and RAW macrophages as early as 6 h following exposure. The cellular responses to MWCNTs were characterized by ROS generation, lysosomal membrane destabilization (LMD), mitochondrial permeability and eventual cell death via the apoptotic pathway. ROS generation appeared to precede the other cellular responses, and increased ROS production was associated with LMD [52]. Another study reported ROS-activated, graphene-induced apoptosis that occurred through the MAPK and TGF-β signaling pathways [14]. A comparison study of cellular toxicity between MWCNTs and onion-like shell-shaped carbon nanoparticles (SCNPs) was conducted on the human bronchial epithelial cell line 16HBE14o-. The presence of NAC, an antioxidant that acts as an ROS scavenger, restored cell viability remarkably, suggesting an association between ROS generation and cellular toxicity [53]. A number of studies have identified oxidative stress as a common mechanism of CNT-induced cell toxicity. SWCNTs induced oxidative stress and cellular toxicity in human epidermal keratinocytes [54], and incubation with SWCNTs triggered ROS generation and consequently cell death along with NF-κB and p38 activation [55]. The addition of dipalmitoylphosphatidylcholine (DPPC) increased the level of cellular ROS, whereas fetal calf serum (FCS) appeared to protect exposed cells against oxidative injury [56]. In response to MWCNT exposure, ROS production in A549 and RAW264.7 cells increased within minutes, peaking within an hour and decreasing after several hours. The dose-dependent cytotoxicity induced by MWCNTs might be closely related to increased oxidative stress [57].

##### Cell autophagy and lysosomal membrane damage

Recently, lysosomal dysfunction emerged as a potential mechanism of nanomaterial toxicity. Additionally, a lysosome-based process known as cell autophagy was recognized as an important pathway of cell death, and a variety of nanomaterials have been demonstrated to induce autophagosome accumulation. Wan et al. shed light on the potential mechanism of carbon nanomaterial-induced cytotoxicity. The investigators measured the formation of autophagosomes via electron microscopy, pEGFP-LC3 transfection and Western blotting, and decreased autophagic degradation was observed. Lysosome disorders were often associated with autophagy dysfunction, and as expected, the researchers confirmed significant lysosome impairment of murine peritoneal macrophages upon SWCNT and graphene oxide exposure using FITC-dextran staining [40]. High-uptake of SWCNHs induced macrophage apoptosis and necrosis associated with lysosomal dysfunction, and internalized SWCNHs were located in lysosomes, as determined by labeling SWCNH with Alexa Fluor 488 and staining lysosomes with lysotracker red. The accumulation of SWCNH in lysosomes induces ROS generation, which in turn triggers lysosomal membrane damage, causing apoptosis and necrosis. Preloaded FITC-dextran (250 kDa) diffusion from the lysosome to the cytoplasm upon SWCNH treatment confirmed lysosomal membrane rupture (LMR) or lysosomal membrane permeabilization (LMP) [51]. Exposure of macrophages to 1 to100 μg/ml CNTs for 24 h or 48 h resulted in dose- and time-dependent apoptotic cytotoxicity. Furthermore, internalized CNTs were found to accumulate in lysosomes, inducing lysosomal membrane permeabilization followed by a release of cathepsins into the cytoplasm, which led to mitochondrial dysfunction and the generation of ROS [58].

##### Pyroptosis

One study investigated an inflammasome-dependent form of cell death induced by CB nanoparticles, identified as pyroptosis. It was characterized by membrane integrity loss and membrane pore formation, causing cell swelling and eventual cell lysis, but the exact mechanism remained unclear. In that study, exposure to CB nanoparticles resulted in inflammasome activation, caspase-1 cleavage and downstream IL-1β release. A caspase-1 inhibitor and a pyroptosis inhibitor both had protective effects on macrophage viability [47].

##### Apoptosis associated with the mitochondrial pathway and scavenger receptors

Acid-MWCNTs and tau-MWCNTs induced apoptosis of RAW264.7 via the mitochondrial pathway and scavenger receptor. Mitochondrial membrane potential decreased and cytochrome c leaked upon MWCNT treatment, and a specific inhibitor of caspase-9 prevented cells from undergoing apoptosis, indicating that the mitochondrial pathway might be involved in the mechanism of MWCNT-induced apoptosis. In addition, a nonspecific inhibitor of scavenger receptors (poly I) and the specific SR-A inhibitor 2F8 could restore MWCNT-treated RAW264.7 cells from apoptotic death significantly [43]. Similarly, pristine SWCNTs (p-SWCNTs) induced apoptosis in RAW264.7 cells through mitochondrial dysfunction, and ATP production decreased in a dose-dependent manner. Additionally, the generation of ROS was enhanced, and apoptosis- and autophagy-related proteins increased [59]. Tau-MWCNTs and raw-MWCNTs produced dose- and time-dependent cytotoxicity in the peritoneal macrophage cell line (RAW264.7) after treatment at different concentrations. Cytotoxicity was involved in the mitochondria-initiated pathway, whereby mitochondrial membrane potential decreased, cytochrome c was released from mitochondria, cytoplasm Ca^2+^ levels increased and ATPase activity was inhibited after MWCNT exposure [60].

##### Necrosis

**Fig. 2:**
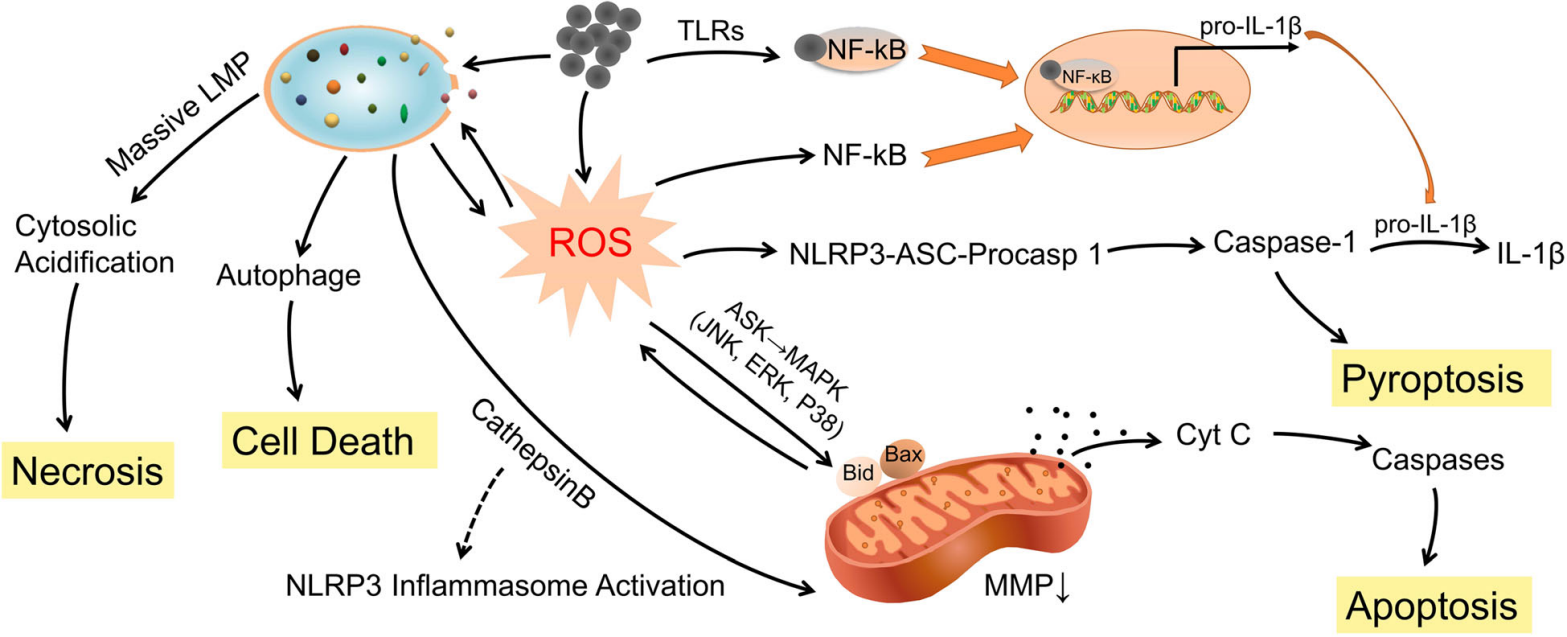
The mechanisms by which carbon-based nanoparticles induce cytotoxicity of macrophages. Exposure of macrophages to carbon nanomaterials triggers a cascade of cellular and molecular events, such as ROS generation and lysosome damage, which serve as the mechanisms underlying carbon nanomaterial-induced cell death, including necrosis, apoptosis and pyroptosis. Carbon nanomaterials cause the mitochondrial dependent apoptotic cascades through ROS-activated MAPKs pathway. ROS could activate several transcription factors, such as NF-κB that regulates the inflammatory response. Carbon nanomaterials induce lysosomal membrane permeabilization (LMP), resulting in the translocation of cathepsins to the cytoplasm. ROS and LMP were reciprocal causation generating an amplification loop. LMP could potentially cause autophagy dysfunction. And inflammasome-dependent pyroptosis was initiated characterized by cleavage of caspase 1 and downstream IL-1β release

It was observed that unpurified MWCNTs entered cells actively and passively by insertion through the plasm membrane, resulting in cell necrosis rather than apoptosis. Decreased cell viability was detected via neutral red staining, MTT assays, confocal microscopy and TEM analysis. Cultures treated with purified MWCNTs (iron catalyst removed) exhibited comparable levels of cell death to those treated with unpurified MWCNTs [61]. In another study, purified SWCNTs induced a low percentage of cell death, apparently necrosis, in human monocyte-derived macrophages (HMMs). The morphology and structure of necrotic cells were observed by TEM images, and large bundles of SWCNTs were ingested by HMMs, localizing in lysosomes or possibly free in the cytoplasm [62]. The mechanisms of carbon nanomaterial-induced cytotoxicity are presented in Fig. 2.

#### Potential factors influencing the cytotoxicity of carbon-based nanomaterials

##### Physical properties of carbon nanomaterials

**Length** Long MWCNTs were cytotoxic to macrophages, whereas short CNTs did not induce any cytotoxicity [63]; additionally, long MWCNTs (20 μm), but not short ones (0.6 μm) significantly decreased cell viability and increased ROS generation in human alveolar macrophages, indicating that MWCNT length is a critical determinant of adverse bioreactivity [64]. Sato et al. evaluated the influence of material length on the cytotoxicity of CNTs against the human acute monocytic leukaemia cell line (THP-1). Two kinds of CNTs with average lengths of 220 nm and 825 nm were prepared. The authors noted that 825-CNTs triggered stronger inflammation than 220-CNTs since the macrophages could envelope 220-CNTs more readily than 825-CNTs [65]. In a toxicity study on short and long CNTs, both were phagocytized by NR8383 rat alveolar macrophages, but long CNTs exerted more significant biological effects, such as cell death, ROS generation and MIP-1α expression. The in vivo results showed that long CNTs induced acute lung inflammation, whereas short CNTs caused slower pulmonary responses [66].

**Size** Nanotoxicological research has demonstrated that the toxicity of nanomaterials is inversely related to particle size. One study assessed the cytotoxicity of MWCNTs of various sizes (diameters < 8 nm, 20-30 nm and > 50 nm; same length, 0.5-2 μm) in 3T3 fibroblasts, bronchial epithelial cells and RAW macrophages. In the study, MWCNTs presented the same mild degree of cytotoxicity in 3T3 fibroblasts as in bronchial epithelial cells, and MWCNTs < 8 nm were more toxic than larger-diameter materials. In contrast, MWCNTs > 50 nm were more toxic than small-diameter materials in RAW264.7 cells [67]. Another study reported that thin MWCNTs (diameter ~50 nm) with high crystallinity and needle-like shape showed mesothelial cell membrane piercing and induced cytotoxicity and carcinogenicity in mesothelial cells. In contrast, thick MWCNTs (diameter ~150 nm) were less toxic, inflammogenic and carcinogenic [68]. Two samples of MWCNTs of different diameters (9.4 vs 70 nm) were prepared. Thinner MWCNTs appeared significantly more toxic than the thicker ones, by evidence of increased LDH activity, ROS production and intracellular GSH depletion [69]. In another study, the authors investigated the biological oxidative effects of carbon black nanoparticles with mean aerodynamic diameters of 14, 56 and 95 nm in rat alveolar epithelial cells and alveolar macrophages. The results showed that smaller particles can induce more prominent oxidative stress in vitro, which may be mediated by the surface function of the particles [32].

**Shape** Carbon nanomaterials with different geometric structures exhibit quite different toxic profiles. A cytotoxicity test protocol for SWCNTs, MWCNTs and C60 was performed to illustrate the influence of different geometric structures of carbon nanomaterials on cytotoxicity. The cytotoxicity of SWCNTs was significantly greater than that of MWCNTs at the same concentration, and both SWCNTs and MWCNTs induced profound toxic effects at a lower concentration, whereas no toxic effect was observed in the C60 group [45]. One study evaluated the toxicity of af-SWCNTs and graphene oxides in primary murine peritoneal macrophages. The results showed that both of the materials induced cell death, autophagosome accumulation and lysosome impairment of macrophages. Although they shared similarities in chemical composition and surface functional groups, graphene oxides were more toxic than af-SWCNTs [40]. The authors claimed that the difference in the toxic potency of graphene oxides and acid-functionalized SWCNTs may be attributable to their distinct physical features. SWCNTs have a tubular shape, tending to penetrate membranes, whereas the flat shape of graphene oxides provides stronger contact with cell membranes, resulting in higher toxicities. In another study [70], the investigators found that the cytotoxicity of differently shaped carbon nanomaterials might not be simply linked to the shapes. The authors found that MWCNTs of elongated shape were more toxic than carbon nanoparticles of spherical shape. The toxicity of carbon nanomaterials might follow the general sequence order as illustrated by relevant studies as follows: fullerenes < carbon black nanoparticles < MWCNTs < SWCNTs < graphene.

##### Surface functionalization of nanomaterials

PEG-functionalized MWCNTs (MWCNTs-PEG) and carboxylated MWCNTs (MWCNTs-COOH) were used to investigate the effect of noncovalent or covalent functionalization of MWCNTs on their cytotoxicity. The results showed that MWCNTs-PEG were less cytotoxic than MWCNTs-COOH. The underlying mechanism was elucidated: lower cellular uptake of MWCNTs-PEG resulted in less generation of ROS and less activation of oxidative stress-responsive pathways such as the p38 MAPK and NF-κB pathways. The study demonstrated that surface functionalization of MWCNTs might alter ROS-mediated cytotoxicity and modulate apoptotic signaling pathways [71].

As previously described, MWCNT carboxylation resulted in damage to the carbon framework and promoted CNT degradation since carboxyl groups combined with associated defect sites, facilitating interaction with lysosomal enzymes [72–74]. Additionally, the damage to the CNTs framework triggered increased degradation in simulated phagolysosomal fluid [75]. A comparative toxicity experiment was performed between acid-purified MWCNTs and concentrated acid-functionalized MWCNTs-COOH [76]. As expected, MWCNTs-COOH yielded less cytotoxicity than MWCNTs, whereas the latter reduced cell viability and increased the release of inflammatory mediators [76]. The binding of human blood proteins to SWCNTs reduced the toxicity of the latter in THP-1 cells and human umbilical vein endothelial cells (HUVECs) [6]. Not only size and dispersion in aqueous solution, but also the degree of functionalization by protein influenced the toxicity of carbon nanomaterials. The cytotoxic effect of CNTs functionalized with a 46-kDa surface protein was evaluated in J774A macrophages. The results showed that functionalized P46-CNTs displayed grade-dependent cytotoxicity, that is, P46-CNTs of high grade were more toxic than those of low grade [77]. Additionally, the nature of the functional protein influenced the cytotoxicity of nanoparticles. Functionalization with even a small amount of the 220-kDa lectin (L220), an immuno-modulatory molecule, decreased the cytotoxicity of CNTs, whereas fluorescein isothiocyanate-functionalized CNTs (FITC-CNTs) presented a greater toxic effect on macrophages, mainly characterized by necrosis [78].

Tau-MWCNTs are MWCNTs that have been modified with taurine on the surface and are more water-soluble than raw-MWCNTs. Cytotoxicity assessment demonstrated that tau-MWCNTs exhibited lower cytotoxicity than raw-MWCNTs [60]. Additionally, it was reported that functionalized SWCNTs that were water-soluble showed reduced cytotoxicity compared to nonfunctionalized SWCNTs in human dermal fibroblasts [79]. Several studies have reported that pristine CNTs are insoluble and induce cell death in vitro. Chemically modified CNTs are highly water-soluble, and their cytotoxicity is associated with their degree of functionalization [45, 79–81].

##### Metal impurities

Manufactured SWCNTs usually contain significant amounts of metal impurities, such as iron, which may act as a catalyst of oxidative stress [82]. Recent studies have shown that metal impurities play a critical role in cytotoxicity and that metal-containing SWCNTs are likely more toxic than metal-free nanoparticles [83]. The purification of CNTs might alter their cytotoxicity, that is, purified CNTs exhibited less toxicity than unpurified ones. However, previous studies demonstrated that the cytotoxic potential was related to the groups added and the purification process applied to the CNTs [78]. In contrast, there is evidence that removing metal catalyst remnants by concentrated acid functionalization does not reduce the toxic potential [53]. Iron catalyst particles generated free radicals through a Fenton-like reaction, leading to lipid peroxidation of the plasma membrane. Thus, SWCNTs purified by acid treatment induced increased toxicity in human monocyte-derived macrophages [62]. It was found that iron-rich (nonpurified) SWCNTs (26 wt.% of iron) presented stronger redox activity than iron-stripped (purified) SWCNTs (0.23 wt.% of iron) because iron might function as a catalyst of oxidative stress, thus causing increased cytotoxicity in RAW264.7 cells [84].

### Immunological Effects of Carbon-based Nanomaterials

Carbon nanomaterials are increasingly being used in medical applications. Different NPs stimulate or interact with various immune cells and different pathways, and the immune response partly depends on the structure and chemistry of the particles [38]. In this section, we highlighted the current findings concerning carbon nanomaterial-mediated immune cell responses in vitro and their immunological effects in vivo (summarized in Table 2).

**Table 2:** In vivo studies on immunological properties of carbon-based nanomaterials

Test materials	Exposure pathways	Exposure doses	Suspension buffer	Time points of sacrifice	Test animals	Main findings	Refe-rence
MWCNTs	Pharyngeal aspiration	40 μg/mouse	PBS with 0.6 mg/mL serum albumin and 0.01 mg/mL 1,2-dipalmitoyl-sn-glycero-3-phosphocholine (DSPC)	24 h, 7 days, and 28 days	C57BL/6 mice aged 10 weeks	An early increase in serum cytokines and inflammatory gene expression in serum; consistently increased eosinophils in blood and BALF.	[34]
Biodegraded and non-biodegraded carbon nanotubes	Pharyngeal aspiration	40 μg/mouse	PBS for non-biodegraded CNTs; PBS with hMPO and H_2_O_2_ for biodegrated CNTs	7 days	C57BL/6 mice	No pro-inflammatory pulmonary response observed in mice treated with biodegraded nanotubes.	[73]
Carbon black nanoparticles	Intratracheal instillation (single)	0.018, 0.054 or 0.162 mg/mouse	0.9% NaCl MilliQ water with 10% (v/v) acellular BAL from C57BL/6 mice.	1, 3 and 28 days	Female C57BL/6 mice aged 5-6 weeks	Strongest lung inflammation on day 1 and day 3 post exposure, elevating for the two highest doses (0.054 and 0.162 mg) 28 days post-exposure.	[114]
High-purity WMCNTs	Orotracheal aspiration	4 mg/kg body weight	PBS with 1% Pluronic F127	1 h, 6 h, 12 h, 18 h, 24 h, 48 h and 72 h	CF-1 Non-Swiss Albino mice aged 6 weeks	Time-dependent neutrophil influx and pro-inflammatory mediator (TNF-α and IL-6) release in bronchoalveolar lavage (BAL) fluid; attenuated influx of neutrophils in AM-depleted mice.	[115]
MWCNTs	Intratracheal instillation; inhalation	0.2 mg or 1 mg/mouse; 0.37mg/m^3^ aerosols (6 h/day, 5 days/week) for 4 weeks	Distilled water with 0.05% Triton X	3 days, 1 week, 1 month, 3 months, and 6 months; 3 days, 1 month, and 3 months	Male Wistar rats aged 9 weeks	Less amounts of MWCNTs delivered into the lungs, and therefore less pulmonary inflammation responses in the group of inhalation exposure compared to intratracheal instillation.	[116]
SWCNTs	Intratracheal instillation	0.04, 0.2, 1 mg/kg body weight	PBS with 10 mg/mL Tween 80	3 days, 1 week, 1 month, 3 months and 6 months	Male Crl: CD (SD) rats aged seven weeks	Increased inflammatory cells in BALF in a dose-dependent manner from 3 days post-exposure up to 3 months; alveolar macrophage accumulation and inflammatory cell infiltration in the lung sections.	[117]
Long tangled MWCNTs and long rod-like MWCNTs	Pharyngeal aspiration	10 or 40 μg/mouse	PBS with 0.6 mg/ml BSA	4 and 16 h or 7, 14, and 28 days	Female C57BL/6 mice aged 7–8 weeks	Attenuated inflammatory reactions caused by CNTs in IL-1R^-/-^mice and antagonist-treated (etanercept and anakinra) mice	[119]
MWCNTs	Pharyngeal aspiration	5, 20, or 40 μg/mouse	Ca^2+^ and Mg^2+^-free PBS with 0.6 mg/ml mouse serum albumin and 0.01 mg/ml DPPC	1, 3, 7 and 14 days	Male C57BL/6J mice aged 8 weeks	Rapid and prominent fibrosis formation remarkably near where the particles were deposited in the lungs; pronounced infiltration of neutrophils and macrophages alongside fibrosis.	[122]
SWCNTs and fullerenes	Intratracheal instillation	Low dose: 0.0003, 0.0015, 0.003, 0.015, and 0.3 mg/mouse; high dose: 0.1 and 0.5 mg/mouse	Pluronic F-68	7 days	Male ICR mice aged 5-6 weeks	Airway hyperreactivity and airflow obstruction; upregulation of cathepsin K, MMP-12, CCL2 and CCL3, and macrophage receptors such as Toll-like receptor 2 and macrophage scavenger receptor 1.	[123]
SWCNTs, MWCNTs and ultrafine carbon black particles	Subcutaneous injection into the footpad together with OVA; intranasal administration together with OVA.	For the injection model, ~200 μg /single dose (max dose with fourfold dilutions) for 3 doses; for the intranasal model, 133 μg/day for 3consecutive days	Hank’s balanced salt solution with 10% serum from BALB/cA mouse	An OVA booster on days 21, sacrificed on days 26; An OVA booster on days 21,22 and 23, sacrificed on days 26.	Female inbred BALB/cAnNCrl mice aged 6–7 weeks	Increased serum levels of OVA-specific IgE, number of eosinophils in BALF, and secretion of Th2-associated cytokines in the mediastinal lymph node in the group of CNTs together with OVA; increased IgG2a levels, neutrophil cell numbers, and levels of TNF-α and MCP-1 in BALF in the group of MWCNT and ufCB with OVA.	[124]
MWCNTs	Intratracheal instillation	50 μg/mouse once a week for 6 weeks	PBS with 0.05% Tween 80	24 h after the final intratracheal administration	Male ICR mice aged 6-7 weeks	Aggravated airway inflammation observed in the group of OVA+MWCNTs, characterized by infiltration of immune cells and production of cytokines; increased levels of serum immunoglobulin (allergen-specific IgG1 and IgE) compared with OVA alone.	[125]
Carbon nanoparticles	Pharyngeal aspiration	2.5 mg/kg body weight	PBS	12 h, 24 h, 48 h	Female Balb/cJRj mice aged 8 weeks	Increased allergic airway inflammation and specific Th2 response in the lymph nodes to OVA in the presence of carbon nanoparticles.	[126]
Carbon nanoparticles	Intratracheal instillation	20 μg/mouse	Pyrogene-free distilled water	3, 6, 12, 18, 24 h, and 3 or 7 days	Female C57BL/6 J mice aged 8–10 weeks	Prominent culmination of neutrophil granulocytes 12 to 24 h after instillation; BAL concentrations and increased levels of neutrophil chemoattractants (CXCL1, -2 and-5) from alveolar epithelial type II cells.	[127]
MWCNTs	Intranasal instillation	administered on days 0, 7, and 14 (75 μg/dose)	A synthetic lung surfactant	On the day 23	Male BALB/cByJ mice aged 9 weeks	Aggravated airway inflammation and the generation of epithelium-derived innate cytokines caused by the coexposure of MWCNTs and HDM compared to HDM alone.	[128]
Rod-like MWCNTs and tangled MWCNTs	Inhalation	6.2-8.2 mg/m^3^ for rCNT and 17.5-18.5 mg/m^3^ for tCNT (4 hours at a time once or on four consecutive days)	—	4 h, 24 h	Female C57BL/6 and BALB/c mice aged 7–8 weeks	Allergic-like airway inflammation and the upregulation of innate immunity-relevant genes and cytokine/chemokine pathways caused by rod-like CNTs after 4-hour exposure.	[129]
MWCNTs	Oropharyngeal aspiration	4 mg/kg body weight	Saline with 10% surfactant	1 or 30 days	C57BL/6J and B6.Cg-kit (W-sh) mast cell deficient mice aged 4–10 weeks	Involvement of mast cells and the IL-33/ST2 axis in the pulmonary and cardiovascular responses to MWCNT exposure; no toxicological effects observed in mast cell-deficient mice or in mice without response to IL-33.	[132]
MWCNTs	Intratracheal instillation	5, 20, and 50 mg/kg body weight	PBS	1,3, 7,14 days	Male ICR mice	Increased pro-inflammatory cytokines in BALF and in blood in a dose-dependent manner, peaking at day 1 postexposure; more pronounced elevation of Th2-type cytokines than that of Th1-type cytokines; considerably enhanced number of B cells in the spleen and blood.	[133]
MWCNTs	Intravenous injection	100 μg/mouse	Ca^2+^ and Mg^2+^-free PBS	24 h, 7 days and 15 days	Female C57BL/6 mice aged 6-8 weeks	Proliferative response of T lymphocytes to a nonspecific mitogen and to ovalbumin (OVA); an increase in expression of proinflammatory cytokines such as TNF-α and IL-6 and IFN-γ, and a decrease in TGF-β and IL-10; increased antibody production to OVA in the treated group.	[134]
nonPEGylated SWCNTs	Intravenous injection	1.2 mg/kg body weight	PBS	6 min and 60 min	Male Wistar rats of body weight 250–280 g	A significant rise in plasma thromboxane B2 levels.	[145]
MWCNTs	Inhalation	0.3, 1, or 5 mg/m^3^ (6 h/day) for 7 or 14 days	—	7 or 14 days	Male C57BL/6 mice aged 10 weeks	An absence of severe inflammation and tissue injury; decreased NK cell function and T cell-dependent antibody response.	[148]
MWCNTs	Inhalation	0, 0.3 or 1 mg/m^3^ (6 h/day) for 14 consecutive days	—	—	Male C57Bl/6 mice aged approximately 8 weeks	A dose-dependent decrease in antibody formation in response to antigen, not altering lymphocyte subpopulations; activation of the cyclooxygenase pathway in the spleen by MWCNTs through TGF-ß release in the lung, leading to T-cell dysfunction and decreased T-cell-dependent antibody formation.	[149]
MWCNTs	Pharyngeal aspiration	50 μg/mouse	PBS with 0.6 mg/ml mouse serum albumin and 0.01 mg/ml DPPC	day 7, 28	Wild-type C57BL/6 and IL-1R^-/-^ mice aged 2 months	Severe acute pulmonary inflammation, and increases of TNF-α, IL-6, IL-1β and MCP-1 protein levels in BALF in wild-type mice; reduced pulmonary inflammatory response in IL-1R^-/-^ mice exposed to MWCNT.	[157]
Four samples of MWCNTs: NT_tang1_ (diameter ~14.84 nm, length 1-5 μm), NT_tang2_ (diameter ~10.40 nm, length 5-20 μm), NT_long1_ (diameter ~84.89 nm, mean length 13 μm) and NT_long2_ (diameter ~165.02 nm, max length 56 μm)	Intraperitoneal injection	50 μg/mouse	Saline with 0.5% BSA	24 h and 7 days	Female C57BL/6 mice aged 8 weeks	Significant polymorphonuclear leukocyte (PMN) or protein exudation and granulomas formation on the peritoneal side of the diaphragm in the group of long-fiber-containing samples.	[160]
Nano-sized carbon black	Inhalation	For a 3-day experiment, 13.08 ± 3.18 mg/m^3^ of non-sonicated carbon black (group N) and 13.67 ± 3.54 mg/m^3^ of sonicated carbon black (group S); for a 2-week experiment, 9.83 ±3.42 mg/m^3^ of non-sonicated carbon black and 9.08 ± 4.49 mg/m^3^ of sonicated carbon black. (exposed 6 h/day, 5 days/week for 3 days or 2 weeks)	Distilled water	—	Male Sprague-Dawley (SD) rats aged 5 weeks	More carbon black particles-laden macrophages observed in BALF and more carbon black deposited in the lungs exposed to sonicated carbon black; no significant difference in the levels of inflammatory cytokines or damage-indicating proteins between the two groups in the 3-day experiment, whereas 2-week exposure induced increased number of total cells, macrophages, and PMNs in the group S.	[163]
SWCNTs, MWCNTs (diameter 10-15 nm), MWCNTs (diameter 20-50 nm) and MWCNTs (diameter 3-10 nm)	Intratracheal instillation	100 mg/rat	NaCl solution with or without 0.5 mg/ml BSA	24 h	Male Sprague Dawley rats of body weight 180-220 g	Remarkably accentuated inflammatory cell infiltration in BALF, and increased the number of CNT-loaded alveolar macrophages, caused by CNTs dispersed in BSA, but not in NaCl solution, indicating the potential importance of CNT dispersion for the toxicological studies.	[164]
Several types of covalently functionalized MWCNTs: COOH-MWCNTs, sw-NH_2_- MWCNTs, NH_2_-MWCNTs, PEG-MWCNTs and PEI-MWCNTs	Oropharyngeal aspiration	2 mg/kg body weight	PBS with or without 0.6 mg/mL BSA and 0.01 mg/mL DPPC	40 h or 21 days	Male C57BL/6 mice aged 8 weeks	Obvious lung fibrosis induced by cationic PEI-MWCNTs, whereas reduced pulmonary fibrosis observed in the group of MWCNT-COOH.	[167]
Tau-MWCNTs and raw MWCNTs	Intratracheal instillation	0.125, 0.25, 0.5 or 1 mg/kg body weight	PBS	1, 7, 14 or 28 days	Male CD-1 (ICR) mice of body weight 18∼22 g	Less toxic induced by Tau-MWNTs than insoluble raw MWNTs	[168]

### Carbon Nanomaterial-mediated Immune Cell Responses in Vitro

#### The effect on macrophages

The majority of the works of carbon nanomaterial-mediated immune cell responses, including the effects on cellular uptake, cellular viability, and induction of inflammation, have been carried out on macrophages. Several studies have suggested that engineered nanoparticles might influence the cellular uptake of macrophages. For instance, shorter CNTs can be engulfed by different immune cells in vitro; however, long CNTs induced the so-called frustrated phagocytosis in macrophages, which triggered persistent inflammatory responses and increased the production of ROS and cytokines [85, 86]. Internalisation of CNTs by immune cells is also a critical determinant in biomedical applications [87]. Macrophages, as typical phagocytes, are very attractive targets for selective drug delivery [88]. Poor recognition of CNTs by macrophages is prohibitive of macrophage-mediated “surveillance” [18, 89], whereas functionalization of CNTs increases their recognition by phagocytes and other cells [80, 90–92]. Apoptotic cells expose an anionic phospholipid, phosphatidylserine (PS), on the cell surface as the “eat-me” signal for recognition and uptake by macrophages [93]. In one study, PS-coated SWCNTs were recognized by different phagocytic cells (i.e., murine RAW264.7 macrophages, primary monocyte-derived human macrophages, dendritic cells, and rat brain microglia), and their uptake was suppressed by the PS-binding protein Annexin V. Additionally, PS coating changed the pro- and anti-inflammatory behaviours of macrophages induced by nanoparticles [88]. Another study demonstrated that phenylboronic acid (PB)-modified carbon dot nanoparticles could be taken up by macrophages with low toxicity and high efficiency compared to unmodified nanoparticles. The findings suggested that these modified nanoparticles are good candidates for delivering drugs to suppress or eliminate aberrant immune cells, such as tumour-associated macrophages [94].

Carbon nanomaterials reportedly exhibit harmful effects on macrophages. Macrophages play a key role in the ingestion of microorganisms and tissue homeostasis. One study revealed that the function of human monocyte-derived macrophages (HMDMs) might be comprised by SWCNTs due to suppressive effects. SWCNTs at non-cytotoxic concentrations suppressed HMDM chemotaxis and impaired the engulfment of apoptotic target cells by macrophages following pre-incubation with SWCNTs [95]. One in vitro study investigated the potential pathophysiological consequences of primary human alveolar macrophages exposed to long MWCNTs (20 μm in length) and short MWCNTs (0.6 μm in length). After a 24-hour treatment with long MWCNTs, alveolar macrophage viability decreased, superoxide levels increased, and inflammatory mediator release increased, and phagocytosis and migratory capacity of alveolar macrophages decreased [96]. In addition to causing cytotoxicity, pristine MWCNTs and functionalized MWCNTs with COOH and PEG reportedly induce significant production of inflammatory cytokines, including TNF-α, IL-1β and IL-6, at different concentrations from 25 to 200 μg/ml [97]. CB, short CNTs, long and tangled CNTs, and long and needle-like CNTs were used to compare their potencies to induce secretion of IL-1 family cytokines. The authors found that long and needle-like CNTs activated the secretion of IL-1β and IL-1α from HMDMs, dependent on NLRP3 inflammasome activation. Moreover, it was noted that ROS production and cathepsin B activation were involved in CNT-induced NLRP3 inflammasome activation [98].

#### The effect on lymphocytes

Lymphocytes are responsible for the antigen-specific and innate characteristics of the immune response. Several reports have elaborated on the positive or negative modulation effects of carbon nanomaterials on the cell function of lymphocytes. One study investigated the impact of functionalized CNTs on T and B lymphocytes and natural killer (NK) cells. The results showed that strong activation was induced in NK cells [99]. In one study, the authors found that CNTs at low doses (0.00-0.1 μg/ml) did not induce cellular toxicity, but they enhanced lymphocyte-mediated cytotoxicity against multiple human cell lines through NF-κB activation in lymphocytes. Additionally, CNTs induced an increase in secretion of IFN-γ and TNF-α from lymphocytes [100]. Another study reported that SWCNTs at concentrations of 1 and 10 μg/ml had no proliferation effects on spleen cells, and SWCNTs at 25 or 50 μg/ml promoted the proliferation of spleen cells. In turn, SWCNTs inhibited T lymphocyte proliferation at higher concentrations and inhibited LPS-induced B lymphocyte proliferation at concentrations of 1, 10, 25 and 50 μg/ml. Moreover, they significantly decreased NK cell activity [101].

Carbon nanomaterial-induced cytotoxicity in lymphocytes was reported. In one study, the authors found that oxidized MWCNTs (400 μg/ml) induced a massive loss of cell viability of human T cells through programmed cell death, whereas pristine MWCNTs were less toxic [102]. In contrast, another study found that two types of functionalized CNTs (the 1,3-dipolar cycloaddition reaction and the oxidation/amidation treatment, respectively) were taken up by B and T lymphocytes and macrophages in vitro, without affecting cell viability [103].

#### The effect on dendritic cells

Dendritic cell-based immunotherapy is an important type of anticancer strategy and has generated promising results in clinical trials [104, 105]. In one study, the authors demonstrated that MWCNTs conjugated to tumour proteins increased the uptake of tumour antigens by human DCs and induced anticancer responses of DCs in vitro [106]. The inflammatory potential of carbon nanoparticles represents a critical issue regarding their biomedical applications. One study explored the inflammatory properties of CNTs and carbon nano-onions (CNOs) in dendritic cells. Highly purified CNTs and CNOs (p-CNTs and p-CNOs) failed to induce dendritic cell maturation or secretion of inflammatory cytokines (IL-6 and TNF-α). However, p-CNTs and p-CNOs promoted the secretion of IL-1β by dendritic cells in a NLRP3- and dose-dependent manner, whereas CNOs exhibited a weaker inflammatory profile. Remarkably, the benzoic acid functionalization of p-CNTs and p-CNOs reduced their inflammatory activity, which was characterized by the reduced secretion of IL-1β [107].

#### The effect on other immune cells

Evidence of carbon nanomaterial-mediated cellular responses in other types of immune cells is insufficient. One study investigated the impact of functionalized CNTs on monocytes in vitro. The results showed that strong activation was induced in monocytes by CNTs [99].

### Immunological Effects of Carbon-based Nanomaterials in Vivo

#### Pulmonary macrophage activation and inflammation induction

Alveolar macrophages, as resident phagocytes in the lung, play a central role in particle removal by nonspecific phagocytosis. The first response of macrophages against inhaled particles is to recognize these particles by cell surface receptors, such as the mannose receptor (MR), the toll-like receptor (TLR) and the scavenger receptor (SR) [108, 109]. MR-mediated phagocytosis results in a variety of downstream events, including the permeability of the lysosome, the generation of ROS, the activation of NF-κB and the release of proinflammatory cytokines, such as IL-1, IL-6, and TNF-α [110–113]. However, SR-mediated phagocytosis is not accompanied by proinflammatory cytokine secretion and NF-κB activation [108].

**Fig. 3:**
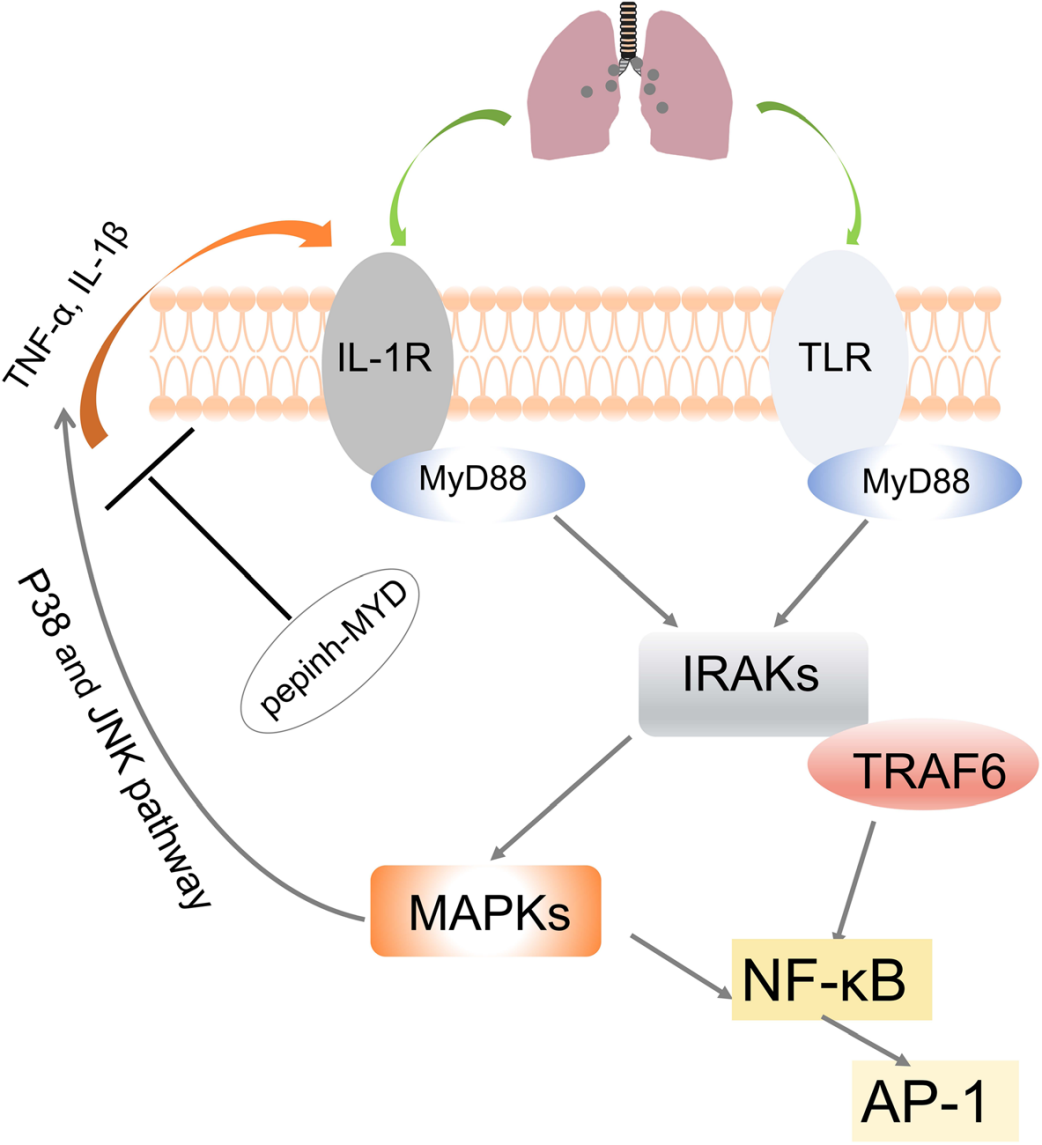
MyD88 played a critical role in alveolar macrophage-mediated inflammatory response to CNTs. MyD88 mediated CNTs toxicity by linking IL-1R or TLR-dependent signaling and acted on downstream IRAKs and TRAFs, thus inducing proinflammatory NF-κB pathway. Also, MAPKs was involved in toxic response and MAPK inhibitors for p38 and JNK reduced levels of TNF-α and IL-1β. MyD88-specific inhibitory peptide blocked the production of TNF-α and IL-1β

After particle internalisation, activated macrophages mediated carbon nanomaterial-induced pulmonary inflammation and fibrotic changes. In the early stage of CNT-induced inflammation (inhalation or intraperitoneal injection), macrophages are the major type of recruited inflammatory cells. After a single intratracheal instillation of carbon black nanoparticles at higher doses (i.e., 0.054 and 0.162 mg), the number of macrophages in bronchoalveolar lavage (BALF) decreased on the first day but elevated on the third day [114]. Frank et al. revealed that exposure to MWCNTs (4 mg/kg body weight) in mice via orotracheal aspiration caused a time-dependent neutrophil influx and proinflammatory mediator release (TNF-α and IL-6) in the BALF at indicated time points. Alveolar macrophages functioned as the major effector cells in CNT-triggered pulmonary inflammation through the MyD88 pathway. The underlying mechanism is presented in Fig. 3. The depletion of alveolar macrophages by liposomal clodronate (LC) pretreatment reduced CNT-induced inflammation in vivo, and further in vitro experiments identified the effector role of alveolar macrophages in inflammatory responses [115].

In the chronic phase after MWCNT exposure (inhalation or intratracheal instillation), macrophages remained in rat lungs as the main infiltrating cells [116]. Intratracheal instillation of SWCNTs (i.e., 0.04, 0.2 and 1 mg/kg) led to increased numbers of macrophages in BALF in a dose-dependent manner from day 3 up to 3 months after exposure. Alveolar macrophage accumulation was observed in the lung sections at day 3 up to 6 months after instillation. Deposited SWCNTs in the lungs were phagocytosed by alveolar macrophages or macrophages in the interstitial tissues, which was related to inflammation and fibrotic changes [117]. Intratracheal instillation of MWCNTs (4 mg/kg) induced pulmonary interstitial fibrosis in rat lungs where the alveolar macrophages produced platelet-derived growth factors (PDGF)-AA, an important mediator of pro-fibrosis [118].

In one study, two different CNTs (long tangled and long rod-like CNTs) and asbestos were used for pharyngeal aspiration (10 μg or 40 μg per mouse) in mice. Sixteen hours after exposure, rod-like CNTs and asbestos induced strong neutrophil influx into the lungs with increased proinflammatory cytokines and chemokines. IL-1R^-/-^mice and antagonist-treated (etanercept and anakinra) mice presented attenuated inflammatory reactions, indicating the important role of the IL-1R signaling pathway in the observed pulmonary inflammation [119]. Chronic exposure resulted in lung pathology such as hyperplasia and granulomatous lesions [120, 121]. MWCNTs (5 to 40 μg) via pharyngeal aspiration promoted rapid and prominent fibrosis formation remarkably near where the particles were deposited in the lungs and could also significantly increase TGF-β1 and PDGF-A in the lungs. Pronounced infiltration of neutrophils and macrophages was observed alongside fibrosis [122].

**Fig. 4:**
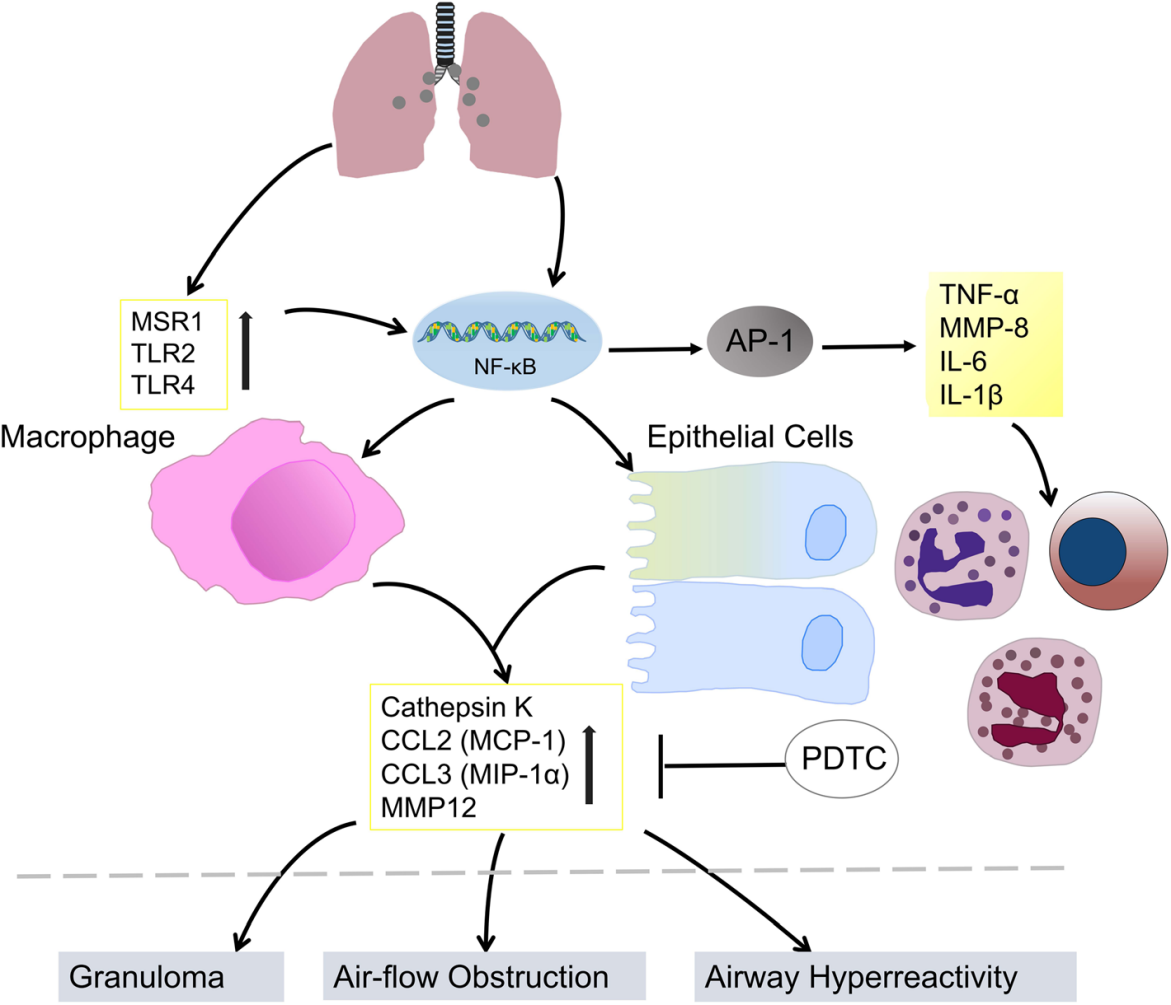
SWCNTs induced lung injury. Inhalation of SWCNTs could up-regulate chemokines, proteinases and several macrophage receptors, also resulting in NF-κB-related inflammatory responses, which play roles in lung pathology including airway hyperreaction, airflow obstruction and granuloma. In vitro experiments indicated that the cell-cell interaction of bronchoalveolar macrophages with lung epithelial cells induced MMP12 and cathepsin K. Blocking NF-κB with PDTC could attenuate SWCNTs-induced chemokine and proteinase expression

Intratracheal instillation of SWCNTs (0.3 μg to 0.5 mg per mouse) induced airway hyperreactivity (AHR) and airflow obstruction, mimicking obstructive airway disease. The underlying mechanism is presented in Fig. 4. Several proteins are upregulated, including cathepsin K, MMP 12, CCL2 and CCL3, and macrophage receptors such as Toll-like receptor 2 and macrophage scavenger receptor 1. SWCNT-induced airway hyperreactivity can be attenuated by a cathepsin K inhibitor. The NF-κB pathway and downstream signals dominate the pathologic process and inflammatory responses. Pyrrolidine dithiocarbamate, an NF-κB inhibitor, reduced the pathologic process as well as MMP12 and cathepsin K expression in animals [123].

In addition to direct effects, carbon nanomaterials enhanced airway allergic immune responses to allergens. After footpad injection and intranasal administration of carbon nanoparticles with an OVA booster, the levels of OVA-specific IgE and IgG1 rose significantly higher than they did after OVA alone. Intranasal exposure induced airway inflammation characterized by increased cell numbers (neutrophils, lymphocytes, and eosinophils) and cytokine levels (MCP-1 and TNF-α) in BALF. Additionally, the total number of cells in the MLNs (mediastinal lymph nodes) was increased in the treated group [124]. With an OVA booster, pulmonary exposure to MWCNTs by repeated intratracheal administration aggravated airway inflammation (infiltration of immune cells and production of cytokines) and increased serum immunoglobulin levels (allergen-specific IgG1 and IgE) compared with allergen alone [125]. In the presence of carbon nanoparticles via pharyngeal aspiration (2.5 mg/kg body weight), exposure to an antigen induced increased allergic airway inflammation and specific TH2 response in the lymph nodes. The application of ectoine reduced these effects during sensitization. The results showed that exposure of the airways to carbonaceous nanoparticles exhibited adjuvant effects by aggravating the allergic immune response during the sensitization against allergens [126].

#### Activation of other immune cells

##### Airway epithelial cells

Shortly after airway exposure via instillation (20 μg), an acute inflammatory response was observed in BALF, characterized by prominent neutrophil accumulation and increased levels of neutrophil chemoattractants. As early as 3 h, 50% of AMs were loaded with black particle, and extracted AMs from the lungs presented no proinflammatory signature. In contrast, through gene expression analysis, alveolar epithelial type II cells were identified as the primary source of CXCL cytokines [127]. A mouse model of asthma was induced using house dust mites (HDM), and the mice were then exposed to MWCNTs by intranasal instillation. The results showed that exposure to multiwalled carbon nanotubes aggravated airway inflammation and induced the generation of epithelium-derived innate cytokines compared with exposure to HDM alone [128].

##### Mast cells

In one study, two different types of CNTs (rigid rod-like and flexible tangled CNTs) were used by inhalation for 4 h/day for 4 consecutive days, mimicking one-week of occupational exposure. The results showed that rod-like CNTs caused allergic-like airway inflammation. Rod-shaped CNTs but not tangled CNTs, promoted the upregulation of innate immunity-relevant genes and cytokine/chemokine pathways after 4 h of exposure. Mast cell-deficient mice presented attenuated lung inflammation, suggesting the critical role of mast cells in allergic inflammation induced by rod-like CNTs [129]. Mast cells make responses to danger signals through various receptors, such as the IL-1-like receptor ST2, which is activated upon combining with its ligand IL-33 [130, 131]. In one study, the mice received a single dose of MWCNTs (4 mg/kg body weight) by oropharyngeal aspiration. For the first time, the study confirmed that mast cells and the IL-33/ST2 axis mediated MWCNT-induced pulmonary and cardiovascular responses. he toxicological effects of MWCNTs were not observed in mast cell-deficient mice or in mice without response to IL-33 [132].

##### Lymphocyte

Intratracheal instillation of MWCNTs (5, 20, and 50 mg/kg body weight) increased proinflammatory cytokines in BALF and in blood, peaking at day 1 postexposure. The elevation of Th2-type cytokines was more pronounced than that of Th1-type cytokines. Additionally, the number of B cells in the spleen and blood was considerably enhanced, indicating B cell activation by Th2-type cytokines [133]. In another study, MWCNTs stimulated the proliferative response of T lymphocytes after intravenous injection (100 μg/mouse), and mRNA expression of proinflammatory macrophage-produced cytokines such as TNF-α and IL-6 significantly increased. IFN-γ, a proinflammatory cytokine, was mainly produced by lymphocytes and the administration of MWCNTs induced immunized mice to release IFN-γ, TNF-α and IL-6, indicating the important adjuvant immune effect of MWCNTs. MWCNTs induced the release of proinflammatory cytokines by macrophages, which in turns stimulates T lymphocytes [134].

##### Systemic activation of immune cells

There is evidence that local exposure (airway exposure or intraperitoneal injection) induces not only local adverse effects, but also systematic inflammatory responses. Pharyngeal aspiration of CNT (40 μg per mouse) caused an early increase in serum cytokines and inflammatory gene expression in serum. Eosinophils in blood and BALF consistently increased beyond 24 h, and after 28 days, systemic markers of immune function were increased [34].

#### Activation of the complement system

After entering the bloodstream, CNTs may interact with the immune system, and the complement system is an integral part of the innate immune system, that is involved in defense against foreign pathogens and in the clearance of debris [135]. Complement activation leads to the production of the anaphylatoxins and chemoattractants C3a and C5a, which may cause anaphylaxis in sensitive individuals [136]. The C1 complex can initiate the activation of the classical complement pathway, triggering a cascade of proteolytic reactions. The C1 complex comprises two subunits: C1q as the recognition protein and C1s-C1r-C1r-C1s as the proenzyme tetrameric complex [137, 138]. One study observed that despite the organized binding of C1q and C1s-C1r-C1r-C1s on carbon nanotubes, no nanotubes induced the activation of the C1 complex [135]. In contrast, Salvador-Morales et al. reported that nonfunctionalized high-pressure carbon monoxide single-walled carbon nanotubes (HiPco SWNTs) and double-walled carbon nanotubes (DWNTs) could trigger the activation of the complement system [139].

PEGylation is widely used for surface modification of nanoparticles, increasing nanoparticle stabilization and prolonging circulation time in the bloodstream [140]. Surface PEGylation of carbon nanotubes makes the nanoparticles dispersible in aqueous solvents, which improves their utility in a variety of applications [141–144]. One study reported that PEGylated nanotubes induced elevation of SC5b-9, and in C1q-depleted serum, nanotubes mediated complement activation, indicating the independence of classical pathway. Intravenous administration of PEGylated nanotubes (1.2 mg/kg) in rats induced a remarkable increase in plasma thromboxane B2 levels, which suggested that nanotube initiated complement activation in vivo [145]. MWCNTs-PEG, despite differing PEG molecular weights, led to increases in serum levels of C4d [145, 146] and SC5b-9 [145] in a concentration-dependent manner, which is indicative of complement system activation in human serum. To shed light on the role of PEG surface engineering in complement activation, one extended study was performed with MWCNTs-PEG of different chain lengths. The results showed that all PEGylated nanotubes were capable of activating the complement system equally, independent of PEG loading length [147]. Albumin-coated SWCNTs were more water-soluble and activated C1q-mediated classical and alternative pathways differently. These findings provide insight into the utility of nanomaterial surface modification to increase innate immunocompatibility [146].

#### Inducing immunosuppression

There were reports that CNTs did not induce inflammatory responses as expected, but even caused immunosuppression. In one study of exposure of MWCNTs by inhalation (0.3-5 mg/m^3^, 6 h/day) for 7 or 14 days, no inflammation or tissue damage was observed. However, MWCNT exposure induced immunosuppression, characterized by decreased NK cell function and T cell-dependent antibody response. Lung histopathology of exposed animals showed alveolar macrophages loaded with black particles, and particle-laden macrophages were also observed in bronchial alveolar lavage fluid [148]. MWCNT inhalation (0.3 or 1 mg/m^3^, 6 h/day) for 14 consecutive days induced compromised systemic immune function in mice, characterized by a decrease in antibody production in response to antigen challenge, but inhaled MWCNTs did not alter lymphocyte subpopulations. Ibuprofen partially rescued exposed animals from T cell-dependent antibody suppression, and cyclooxygenase-2 knockout mice were insusceptible to MWCNT-induced immunosuppression [149].

#### Biodegradation of carbon nanoparticles by immune cells

A comparative study was performed to evaluate the capacity of different peroxidases (HRP, MPO, LPO) to degrade SWCNTs, and the results from an in vitro model showed that three oxidants reacted with SWCNTs, causing their degradation [150]. Myeloperoxidase (MPO) and lipid peroxide (LPO) presented a higher capacity of degrading SWCNTs, attributed to the formation of hypohalous acid (HOCl and HOBr, respectively) [151, 152]. MPO are produced by activated neutrophils; therefore, MPO and HOCl may be present at high concentrations at inflammatory sites, favoring the degradation of nanotubes [153]. Shvedova et al. (2012a) found that the clearance of SWCNTs from the lungs of exposed MPO knockout mice was remarkably reduced compared with clearance in wild-type animals, indicating the involvement of MPO in the biodegradation of SWCNTs in vivo [154]. It has been proposed that the biodegradation of carbon nanotubes by MPO might reduce pulmonary inflammation. show $132#?>inflammatory response. In contrast, the number of neutrophils and the levels of cytokines in BALF after pharyngeal aspiration of biodegraded nanotubes were indistinguishable from those in the control group. The findings indicated that inflammatory responses induced by carbon nanotubes were heavily dependent on the degree of biodegradation of particles in exposed individuals [73].

Eosinophil peroxidase (EPO), as a heme-containing haloperoxidase, is one major enzyme-generating oxidant in the human lung, notably at inflammatory sites and has 68% sequence identity to MPO [155]. The superoxide-generating activity of eosinophils is robust due to the high expression of NADPH oxidase [156]. It was reported that CNT exposure via pharyngeal aspiration (50 μg/mouse) could induce acute pulmonary eosinophilia and the subsequent release of EPO by eosinophils into inflammatory sites in the lungs [157]. Another study found that EPO released from eosinophils mediated the biodegradation of SWCNTs, which suggested that activated eosinophils played an important role in pulmonary responses to these materials [158].

#### Potential factors influencing immunological effects in vivo

#### Physical properties of carbon nanomaterials

##### Length

Two types of SWCNTs, namely relatively thin bundles with short linear shapes (CNT-1) and thick bundles with long linear shapes (CNT-2), were prepared for in vivo tests in rats. The results showed that CNT-2 induced acute lung inflammation shortly after exposure, suggesting that SWCNT-induced pulmonary toxicity is closely associated with the length of particles [66]. CNTs reportedly elicit a length-dependent inflammatory response in the pleural cavity of mice. The authors addressed that macrophages were activated by long fibres via frustrated phagocytosis that stimulated a dramatic amplification of proinflammatory cytokines from mesothelial cells [159]. In another study, the results showed that peritoneal exposure to CNTs resulted in asbestos-like, length-dependent inflammation and granuloma changes. Only the samples containing long fibres induced significant polymorphonuclear leukocyte (PMN) and protein exudation. Granuloma formation was observed on the peritoneal side of the diaphragm in mice after treatment with long fibres [160].

##### Size

Two samples of MWCNTs with similar lengths but different diameters (9.4 vs 70 nm) were intratracheally instilled in rats. The thinner MWCNTs induced greater LDH activity and infiltration of lymphocytes and neutrophils in BALF than the thicker MWCNTs [69]. Ultrafine particles are important contributing factors in the adverse health effects of particulate air pollution. One study compared the lung toxicity induced by ultrafine carbon black (ufCB) and carbon black particles with mean diameters 14.3 nm and 260.2 nm, respectively. As a result, ufCB induced more neutrophil recruitment and epithelial injury than fine particles in rat lungs after instillation at an equal dose [161]. In another similar study, the toxic effects of ufCB (diameter 14 nm) and CB particles (diameter 320 nm) were tested. UfCB induced more prominent neutrophil recruitment and LDH activity in BALF than CB after 24 h of instillation [162]. Sonication is often used to deagglomerate carbon nanoparticles, and one study investigated the effect of sonication on toxic responses. Rats were exposed to carbon black aerosols with (group S) or without (group N) sonication. Compared to group N, group S had a notable increase in the number of particles deposited in the lungs and in the amount of inflammation. No differences were observed in the levels of inflammatory cytokines or damage-indicating proteins between the two groups in the 3-day experiment, whereas 2-week exposure induced increased numbers of total cells, macrophages, and PMNs in group S [163].

##### Shape

Two different types of CNTs (rigid rod-like and flexible tangled CNTs) were used by inhalation, mimicking one-week of occupational exposure, and the results showed that rod-like CNTs caused allergic-like airway inflammation. Rod-shaped CNTs but not tangled CNTs, promoted the upregulation of innate immunity-relevant genes and cytokine/chemokine pathways after 4-hour exposure [129].

##### Dispersion

CNTs dispersed in BSA, but not in NaCl solution, remarkably accentuated inflammatory cell infiltration in BALF, and the number of CNT-loaded cells was also increased [164]. A recent report might provide a possible explanation. The study showed that CNTs presented improved dispersion in media containing FCS due to the physisorption between CNTs and the protein in the FCS-media through van der Waals forces. Similarly, BSA improved CNT dispersion without surface modification, providing new insights into the biocompatibility and toxic effects of nanoparticles [165].

##### Surface functionalization of nanomaterials

MWCNTs and MWCNTs-COOH were tested in mice for pulmonary inflammation and injury biomarkers. The results showed that MWCNTs-COOH presented reduced bioactivity and pathogenicity. Additionally, activation of the NLRP3 inflammasome was found to be involved in MWCNTs- and MWCNTs-COOH-induced pulmonary pathogenicity [166]. Functionalized carbon nanotubes (f-CNTs) are more widely used than unmodified CNTs due to higher dispersion and outstanding physicochemical properties. In one study, several types of covalently functionalized multiwall carbon nanotubes (f-MWCNTs) were used in animals, including carboxylated (COOH), polyethylene glycol (PEG), amine (NH2), sidewall amine (sw-NH2) and polyetherimide (PEI) modified MWCNTs. Compared to pristine MWCNTs, cationic PEI-MWCNTs induced obvious lung fibrosis, whereas reduced pulmonary fibrosis was observed in the group of MWCNT-COOH.These results indicated that surface charge might play an important role in f-MWCNT-induced lung damage [167]. Biochemical and cellular indicators of lung damage were evaluated after exposure to water-soluble tau-MWNTs, raw-MWNTs and positive control crystalline silicon dioxide particles. The findings showed that silicon dioxide particles induced heavier damage to lungs than CNTs and that tau-MWNTs produced slight, reversible pulmonary inflammation in mice. Tau-MWNTs are less toxic than insoluble raw MWNTs, suggesting that SWCNT-induced pulmonary toxicity is closely associated with the solubility of particles [168].

##### Metal impurities

Nine different well-characterized MWCNT were examined in vivo, and they exhibited differential magnitudes of inflammatory and pathogenic potency. The authors pointed out that the outcome was closely related to nickel contamination on the particle surface, and the underlying mechanism might be that the Ni-contaminated particles induced NLRP3 activation followed by cytokine release, resulting in prolonged inflammation and lung pathology [169].

## Conclusions

Due to their unique physicochemical properties, carbon nanomaterials are used for widespread applications ranging from industry to biomedicine. In parallel, carbon nanomaterial exposure has also raised concerns over health hazards associated with their properties [66]. At the beginning of research and development of any carbon nanomaterial, it is necessary to evaluate the potential toxic effects of the material and identify the physicochemical properties that are responsible for toxicity. Additionally, it is crucial to clarify the molecular mechanisms of carbon nanomaterial-induced cytotoxicity [42].

Here, we reviewed the cytotoxicities of carbon nanomaterials in cell culture-based assay systems; however, findings on the topic remain controversial. Some investigators have reported cytotoxic effects against various cell types. In contrast, several studies observed contradictory cytotoxicity results in the same materials and cell types. Despite progress in analytical techniques, assessing the cytotoxicity of carbon nanomaterials still poses considerable challenges. It is not yet understood which aspects of carbon nanomaterials, e.g., surface areas, mass concentrations, lengths, dispersibilities, metal impurities, a combination of these features, or some other factors, play a central role in cytotoxicity. Current toxicological studies have not truly revealed any new cellular toxicity mechanisms of carbon nanomaterials.

Lung epithelial cell lines, such as A549, and macrophage cell lines, such as NR8383, J774, and RAW264.7, are commonly chosen to evaluate the cytotoxicities of carbon nanomaterials. As a lung adenocarcinoma-derived cell line, A549 cells are endowed with cellular markers of type II pulmonary epithelium, serving as a useful in vitro model for studying the biological effects of particulate matter. Multiple types of macrophages are well-characterized cellular models because macrophages phagocytose particles, secrete specific cytokines, synthesize lysozyme and express Fc receptors. Cellular assays showed that carbon nanomaterials generally present more toxicity in macrophages than in other cell types, such as A549 cells, which is consistent with the nature of macrophages as professional phagocytes.

Discussions of toxicity point to poor dispersion of nanomaterials in aqueous media, which challenges the reproducibility of data acquired in toxicological assays [3]. The degree of dispersion of carbon nanomaterials impacts their in vitro and in vivo toxicity. Noncovalent functionalization with surfactants can disrupt bundles of CNTs, thus inducing greatly varied cellular responses. BSA, a water-soluble protein, can be absorbed on the CNT surface and provides excellent dispersing capability for CNTs. In previous studies, BSA, Pluronic F68 (a surfactant) and TWEEN 80 were used for dispersing CNTs in an aqueous environment; as a result, diminished toxic effects were observed [170]. It was reported that CNT-exposed endothelial cells did not exhibit significant acute toxicity in medium containing serum [3]. Similarly, studies on silica toxicity revealed that a synthetic surfactant protected alveolar macrophages *in vitro* against cell death and alleviated lung injury following silica exposure in vivo [171].

Catalytic metals are used during the manufacture of CNTs, and metallic impurities inevitably remain in CNTs. Numerous studies have proposed that residual catalytic metals induce oxidative stress, resulting in cell death. However, a meta-analysis of nanotoxicity studies showed that CNT-induced lung injury is not dominated by metallic impurities [66], and several studies identified the CNTs themselves rather than metal catalysts as the primary cause of cytotoxicity in macrophages [170].

Taurine-functionalized SWCNTs with greater water solubility presented less cytotoxicity against macrophages in some studies [60, 79]. In contrast, other studies found the opposite results. For instance, it was reported that SWCNTs functionalized with carboxylic acid had higher toxicity than pristine SWCNTs in human endothelial cells [172]. The oxidation process reduces the length and straightens the shape of the tubes; thus, it was reported that oxidized MWCNTs induced stronger toxicity than pristine MWCNTs [102]. The difference may be attributed to the chemical and physicochemical parameters of functionalized nanomaterials, such as size, shape, and agglomeration.

In nanotoxicology studies, fluorescent probes are widely used to mark cell death, oxidant production or protein changes. Carbon nanoparticles with surface areas from 20 to 200 m^2^/g serve as universal sorbents of organic compounds in dispersing medium, including not only fluorescent dyes but also proteins, DNA and even salts that are used in toxicity assays. Monterio-Riviere et al. proposed that carbon nanoparticles might interfere with fluorescent assays via absorption or other methods [3]. Indeed, carbon nanomaterials have been found to interfere with assay components and read-out, causing inconsistent results concerning toxicity.

As the application of carbonaceous nanomaterials expands, the size of the exposed population continues to increase and some crucial issues should be addressed regarding their toxicity. Carbon nanomaterials present significantly different cytotoxicity depending on their physicochemical properties, including size, length, shape, and surface area. Additionally, most CNTs are complex mixtures containing multiple carbon forms and catalytic metal residues, which affect the biological cellular responses of exposed cells. Thus, when carbon nanomaterials are tested, it is necessary for researchers to characterize them in detail for the reliability, reproducibility and comparability of data acquired in toxicological assays. In terms of toxicity models, comprehensive experimental information is required to be provided, including the target cell types, dispersion methods, exposure dosage, administration route in vivo [3]. Carbon nanomaterials may present distinct toxic effects on macrophages in different viability assays, since interferences and disturbances are likely to occur. Great care should be taken when carrying out toxicity assays in the presence of fine carbon, and we note that multiple individual cellular bioassays can be performed simultaneously to confirm the findings. Based on comprehensive toxicological studies, better material characteristics are associated with less toxic effects. Differing from many other toxicants, carbon nanomaterials are mostly manufactured; thus, it is practicable for material scientists to modify specific material features, e.g., by removing metal impurities, applying surfactant coatings, or controlling the length of nanotubes to pave the way for possible design of less toxic materials.

It is now clear that the immune system can respond to CNTs and that the interactions are influenced by many factors. Additionally, different types of carbon nanoparticles present different immune compatibility. Physicochemical characteristics of the nanomaterials, such as their lengths, purities, solubilities and surface groups, significantly affect immune system responses. For example, “good” CNTs induce only slight inflammation and organismic damage and have relatively good biocompatibility in nanomedicine applications. Previous studies indicate that “good” CNTs should be short, functionalized, highly water-soluble and readily subjected to oxidative biodegradation. However, intentional or occupational exposure to “bad” CNTs might lead to harmful immune and nonimmune responses. In the future, carbon nanomaterials should be purposefully engineered for low toxicity. On the other hand, based on the immunostimulatory properties of CNTs, carbon nanomaterials could also be engineered for use in vaccines or any therapeutic protocol that requires activation of the immune system .

## Abbreviations

acid-MWCNTs: Acid-treated multiwalled carbon nanotubes
af-SWCNTs: Acid-functionalized single-walled carbon nanotubes
AMs: Alveolar macrophages
BALF: Bronchoalveolar lavage fluid
CB: Carbon black
CNTs: Carbon nanotubes
EPO: Eosinophil peroxidase
FCS: Fetal calf serum
f-MWCNTs: Functionalized multiwall carbon nanotubes
LDH: Lactate dehydrogenase
LMD: Lysosomal membrane destabilization
LPO: Lipid peroxide
MPO: Myeloperoxidase
MRI: Magnetic resonance imaging
MWCNTs: Multiwalled carbon nanotubes
MWCNTs-COOH: Carboxylated multiwalled carbon nanotubes
MWCNTs-PEG: PEG-functionalized multiwalled carbon nanotubes
NG: Nanographite
NPs: Nanoparticles
p-SWCNTs: Pristine single-walled carbon nanotubes
ROS: Reactive oxygen species
SWCNHs: Single-walled carbon nanohorns
SWCNTs: Single-walled carbon nanotubes
tau-MWCNTs: Taurine-functionalized multiwalled carbon nanotubes
